# Engineering Concentration‐Dependent Intravitreal Mobility via Cyclic Arginine‐Enriched Nanocarrier Surface Modification: In Vivo Proof‐of‐Concept in a Porcine Large Animal Model

**DOI:** 10.1002/adhm.71531

**Published:** 2026-08-05

**Authors:** Maximilian Hammer, Bryan Calder Ackermann, Anna Stoß, Niklas Junker, Ludwig Geisweid, Sabrina Wohlfart, Jonathan Herth, Simon William Pohl, Manuel Ben Böhmann, André Schulz, Victor Aristide Augustin, Gerd Uwe Auffarth, Philipp Uhl

**Affiliations:** ^1^ David J Apple Laboratory for Vision Research and University Eye Clinic Heidelberg Heidelberg Germany; ^2^ Faculty of Biosciences Heidelberg University Heidelberg Germany; ^3^ Department of Nuclear Medicine Heidelberg University Hospital Heidelberg Germany; ^4^ Department of Ophthalmology Rostock University Medical Center Rostock Germany; ^5^ Department of Pharmaceutical Technology and Biopharmacy Institute of Pharmacy and Molecular Biotechnology Heidelberg Germany

**Keywords:** intraocular drug delivery, liposomes, retinal disease, surface engineering, vitreous haze

## Abstract

Nanoparticulate drug delivery systems represent a promising platform for sustained intraocular drug release following intravitreal administration, leveraging low metabolic turnover of the vitreous to maintain therapeutic drug levels over extended periods. Despite this potential, nanoparticle diffusion toward the visual axis can induce light scattering and visual disturbance. Given the negative charge of the vitreous, surface‐engineered nanoparticles provide a strategy to modulate particle mobility and restrict off‐target migration. Here, we performed a 6‐week in vivo evaluation in pigs of an intravitreally applied, positively charged, cyclic‐arginine surface‐functionalized liposomal nanocarrier designed to reduce intravitreal mobility. Three formulations containing 0.1%, 0.5%, or 1% cyclic‐arginine‐modified phospholipid were compared with unmodified control liposomes in a large animal pig model. A multimodal biocompatibility and performance assessment was conducted, including intraocular pressure monitoring, fundus imaging, structural and angiographic OCT, dye‐based angiography, and post‐mortem retinal immunostainings. Cyclic‐arginine surface functionalization of the liposomes resulted in a concentration‐dependent reduction in intravitreal mobility, quantified by vitreous haze and fundus‐based distribution analyses. All surface‐engineered nanoparticles demonstrated excellent ocular biocompatibility without structural or vascular adverse effects. These data establish cyclic‐arginine surface modification as a robust design principle to modulate intravitreal mobility and support the development of next‐generation nanoparticle biomaterials for long‐acting ophthalmic drug delivery.

## Graphical abstract

In this study, Hammer et al. highlight cyclic‐arginine surface engineering as a highly biocompatible, controllable, and concentration‐dependent regulator of intravitreal nanoparticle mobility in vivo in a translational large animal model. The cyclic arginine surface‐modification supports the development of next‐generation biomaterials for long‐acting ophthalmic drug delivery.

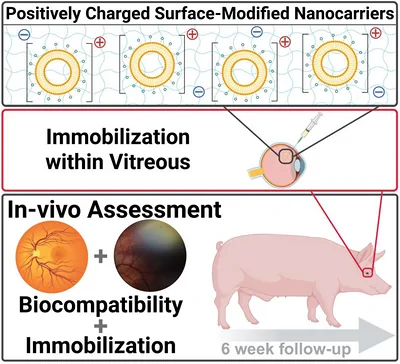

## 1 Introduction

Since their early beginnings in the 1970s, intravitreal injections (IVIs) have become a standard treatment option for a wide range of retinal diseases [1, 2, 3, 4]. As opposed to systemic administration, hindered by the blood‐retina barrier, quick renal clearance, and missing vascularization of the vitreous, IVIs allowed for direct drug application significantly advancing treatment [5, 6]. While IVIs improved treatment options, an even longer intraocular residence time for active compounds, specifically small molecules [7, 8, 9, 10], is desired. One option to slow down clearance represents the encapsulation of drugs in liposomal nanocarriers [11, 12, 13], creating one further diffusion barrier and allowing for controlled, slower drug release [14, 15, 16, 17, 18]. While already well established for intravenous drug administration [19, 20, 21], IVI of liposomal drug formulations has not yet reached common clinical practice [22, 23], though studies in animal models have demonstrated prolonged drug half‐life, decreased toxicity [16, 17, 18] and even further advancements with case reports of first‐in‐human applications [24, 25, 26]. However, patients reported very bothersome bright or dark floaters by liposomes diffusing from the injection site at the pars plana toward the macula, the central area of the retina, located along the optical axis and responsible for the highest visual acuity [24, 25]. This optical limitation of nanoparticles has since hindered their continued application.

At the same time, the vitreous cavity of the eye is filled with the vitreous body [27], a natural hydrogel formed by collagen and glycosaminoglycans [28, 29], specifically high‐molecular weight hyaluronic acid (HA), resulting in a high net‐negative charge of the vitreous body [30]. Thus, surface charge and interfacial chemistry can be applied to modulate the mobility of nanoparticles in this polymeric hydrogel. Therefore, this electrostatic interaction between liposomal nanoparticles and HA‐rich networks could be tunable via surface modification of the particles, an effect achieved by modifying the surface of, for example, liposomes using arginine‐rich peptides (ARPs), in our study more specifically a cyclized nona‐arginine‐modified phospholipid. Modification of liposomal surfaces with such cyclic‐arginine motifs has already been used to optimize efficacy of drug administration by increased penetration of cell barriers [31, 32, 33, 34, 35] and was demonstrated to exhibit strong, multivalent interaction in biological matrices, increasing affinity and reducing diffusion prior breakdown via their property of a high positive surface charge [36, 37, 38, 39]. Although such surface‐modified liposomes have been studied when applied as an intravenous infusion [31, 32, 33, 34, 35, 36, 37, 38, 39, 40, 41], to date, there is no data available when applied intravitreally, especially not in vivo in a translational large animal pig model and further not for their capacity to modulate particle mobility in the vitreous body. However, in vitro or vitreous models lack human‐like vitreous biomechanics and possible inflammatory or cytotoxic reactions [42]. Here, we investigated cyclic‐arginine surface‐modified liposomes at three different concentrations of the surface modification compared to unmodified liposomes and sham‐injections of balanced salt solution (BSS) on their intravitreal mobility and biocompatibility in a large animal pig model over the course of a clinically relevant time frame of 6 weeks (Figure 1). Further, specialized optical coherence tomography (OCT) analyses were applied to monitor the central vitreous haze quantitatively over the whole study period.

**FIGURE 1:**
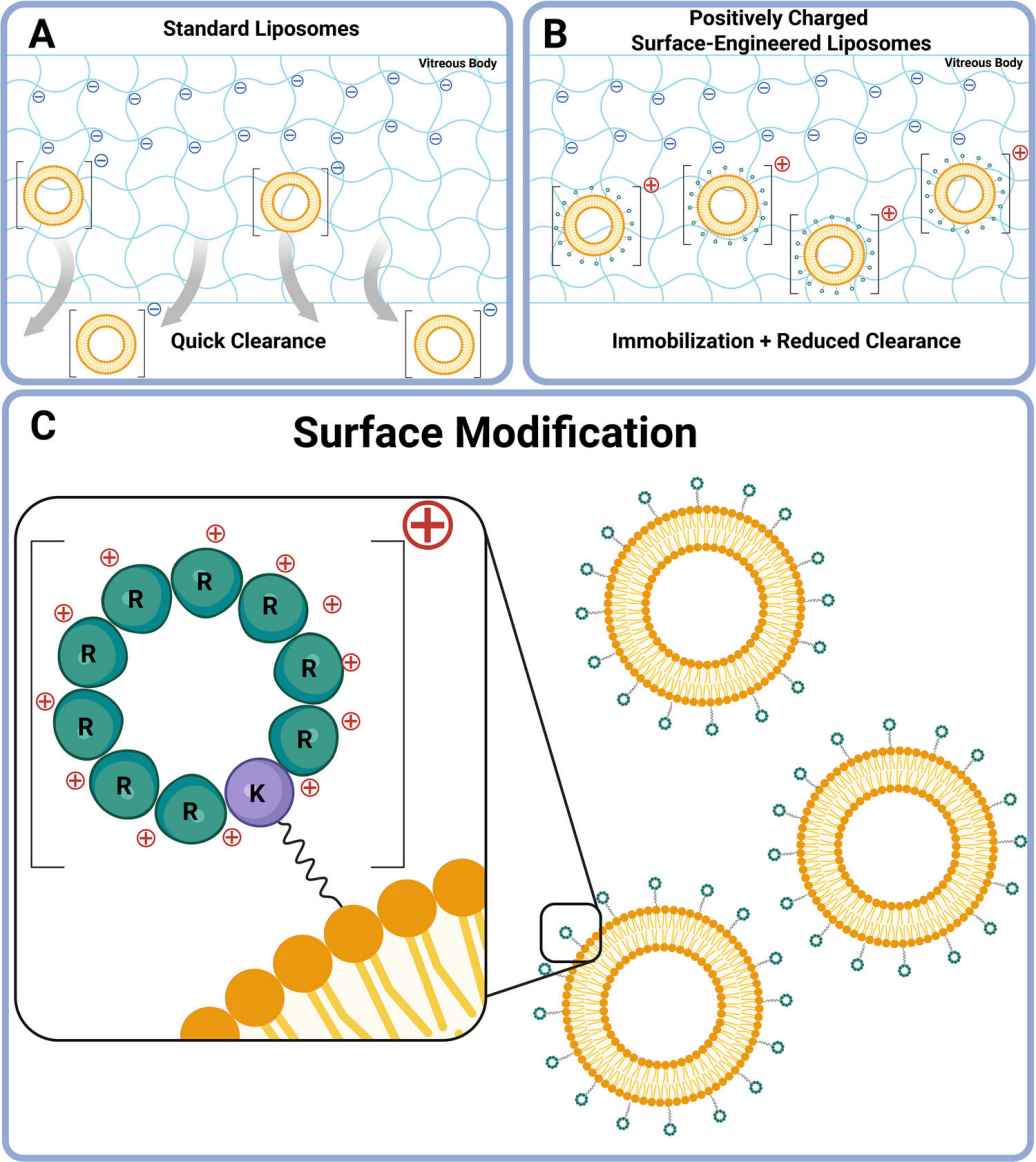
Modulating intravitreal nanoparticle mobility via surface engineering. Standard liposomes show a negative zeta potential, resulting in swift distribution within the vitreous without any immobilization (A). Arginine‐rich, surface‐modified liposomes are positively charged, leading them to be immobilized within the negatively charged vitreous. In consequence, clearance and distribution are reduced depending on degree of the surface modification (B). The liposomal nanocarriers studied have been modified with a cyclic, arginine‐rich peptide on their surface, resulting in a net positive charge (C).

## 2 Results

### 2.1 Liposome Characterization

Liposomal formulations surface‐modified with 0.1%, 0.5%, and 1% ARP‐conjugate as well as control liposomes without surface modification were prepared by dual centrifugation as preparation technique [14]. All liposomal formulations were characterized using dynamic light scattering to determine size and PDI followed by zetapotential determination (Figure 2A,B). With increasing amount of surface modification, the zeta potential and thus the surface charge of the liposomes turned into net positive, reaching mean values of 14.17 mV at 1% ARP surface conjugation. Importantly, size and polydispersity index remained unchanged. Information on composition and particle characteristics is presented in Table S1. Upon incubation with 2% HA (11 kDa), the zeta potential reversed toward negative values, indicating adsorption of HA onto the liposomal surface. The zeta potential for ARP 1% decreased up to a minimum of −12.39 ± 2.80 mV (Figure 2C). All liposomal formulations were examined using Cryo‐TEM demonstrating low lamellarity (Figure 2D shows the ARP 1% formulation as the most prominent example, other formulations are presented in Figure S1).

**FIGURE 2:**
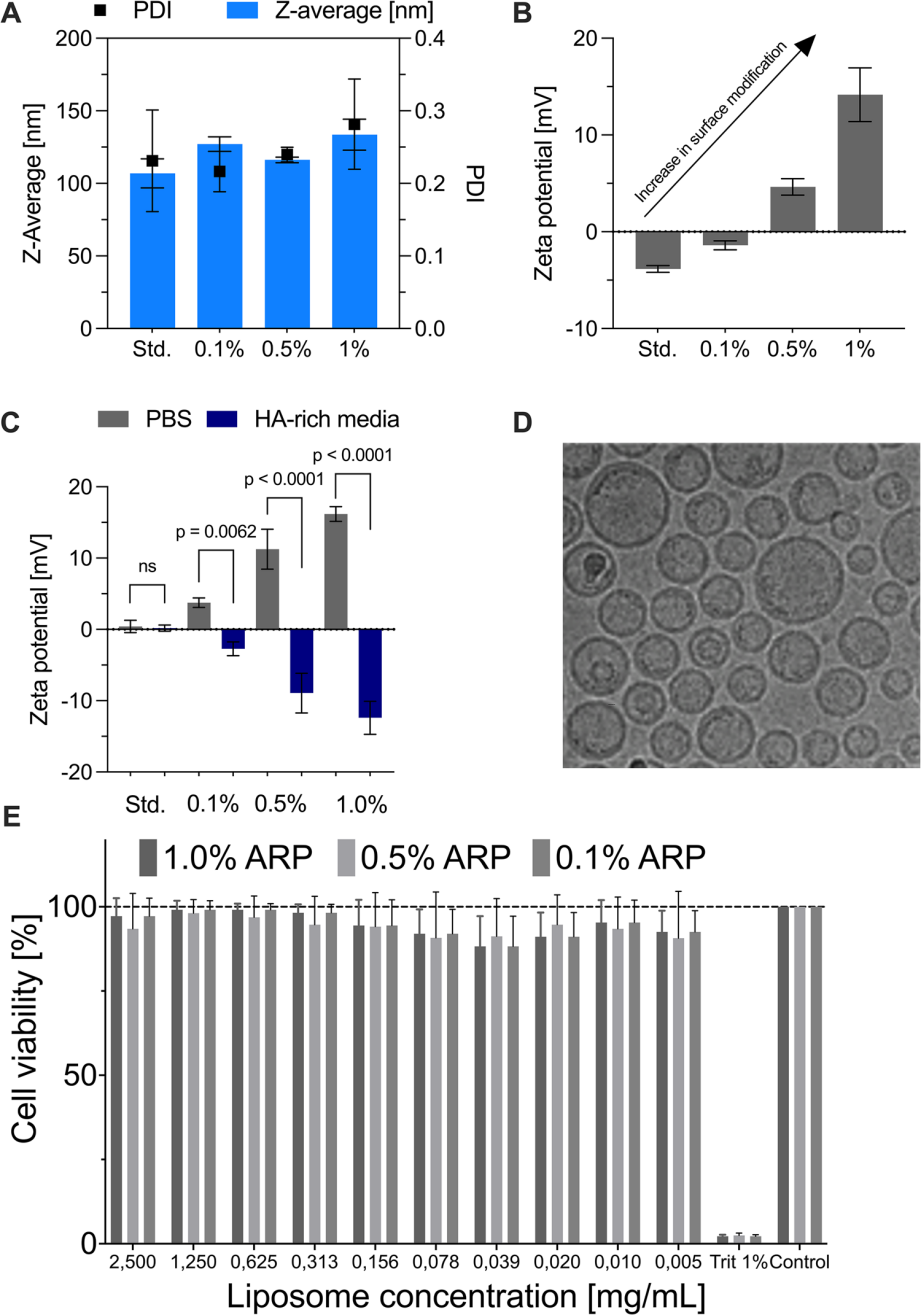
In vitro characterization of liposomal formulations. Polydispersity index (PDI), particle size (A) and zeta‐potential (B) of all liposomal formulations. While size and PDI remain unchanged (A), zeta‐potential turns net positive with increasing ARP surface modification (B). The zeta‐potential increases with higher ARP surface modification, shifting toward positive values in PBS (10%) and toward negative values upon incubation in HA (2%, 11 kDa) (C). Exemplary Cryo‐TEM image of the 1%‐ARP surface modified liposomes showing uniform size and mostly unilamellar and univesicular formation (D). All surface‐modified liposomal formulations showed excellent biocompatibility in ARPE‐19 human pigment epithelium cells (E). Data for (A), (B), (C) & (E) shown as mean ± SD (*N* = 3, *n* = 3).

### 2.2 In Vitro Cytotoxicity Assessment

All liposomal formulations showed excellent biocompatibility on ARPE‐19 cells, an immortalized human retinal pigment epithelium cell line for all tested, clinically relevant, concentrations ranging from 0.005 to 2.5 mg/mL. Results are presented in Figure 2E.

### 2.3 In Vivo Biocompatibility

Biocompatibility of the surface‐ and non‐surface‐modified liposomes was evaluated in a large animal porcine model over 6 weeks. The study procedures are presented in Figure 3A. Animals were examined using an in‐depth biocompatibility evaluation prior to, 2, 4, and 6 weeks after injection of the respective liposomal formulation. Fundus photographs were additionally taken 1, 2, and 3 days after injection to visualize early distribution patterns. Table 1 depicts the formulations tested in individual animals. Additionally, three animals were injected with BSS, to better illuminate possible effects of the intravitreal injection itself on vitreous haze and biocompatibility parameters. According to the 3R‐rules, these control animals’ data was partially presented previously by our group [14, 43]. All animals showed excellent biocompatibility. Specifically, during fundus examination no signs of vessel sheathing, neovascularization, and no atrophic changes to the retina or the optic disc were observed (Figure 3B). Retinal layers remained intact as visualized by OCT (Figure 3C) and mean retinal thickness in the visual streak remained unchanged throughout the study period (no significant influence of treatment or treatment/time interaction, *p* = 0.95 and *p* = 0.16, respectively, REML mixed‐effects model, Figure 3D) and intraocular pressure (IOP) within normal limits (10–16 mmHg) for all animals at all time points (Figure 3E). Electroretinography (ERG) amplitudes and latency (Figure 3F,G) showed no relevant changes indicating uncompromised retinal function even to very low‐light stimuli, detailed ERG analyses by group are presented in Figure S2 again proving no functional impairment of the retina for all formulations. Fluorescein (FAG) and indocyanine angiography (ICG) revealed no signs of vasculitis, localized leakage, or ischemia (Figure 3H).

**FIGURE 3:**
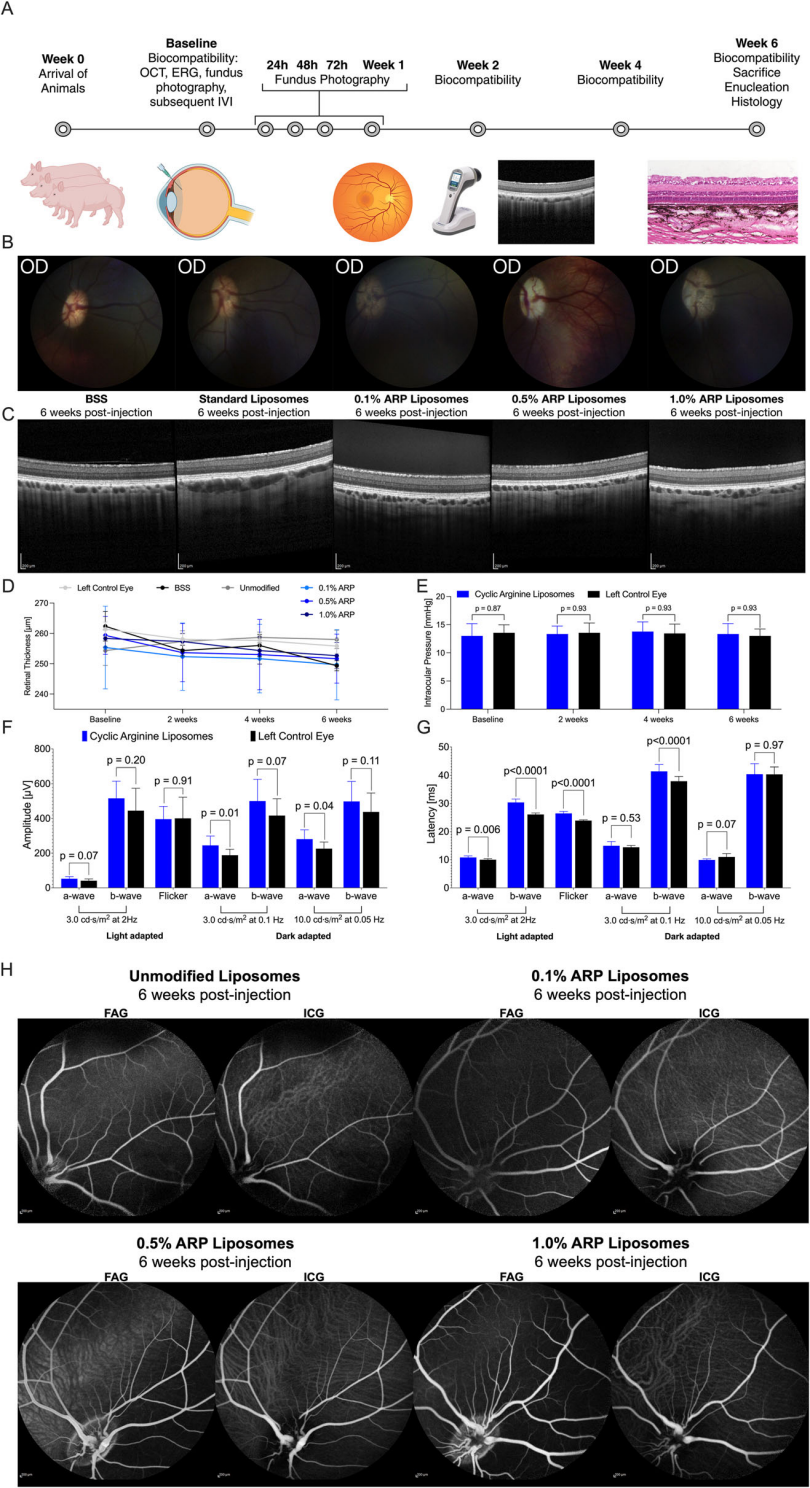
In‐depth in vivo biocompatibility analysis. After an adaptation period of 1 week, animals were anaesthetized and received multimodal biocompatibility examinations. Subsequently, an intravitreal injection was performed with either the surface‐engineered ARP‐liposomes, unmodified liposomes, or with BSS. Biocompatibility examinations were repeated 2, 4, and 6 weeks after the initial injection. At 6 weeks, animals were euthanized, and post‐mortem histological analyses were performed. Additionally, fundus photographs were taken 1, 2, 3, and 7 days after the injection to visualize early particle movement (A). All tested solutions (in total, *n* = 15, unmodified liposomes *n* = 3, 0.1% ARP‐modified *n* = 3, 0.5% ARP‐modified *n* = 3, 1% ARP‐modified *n* = 3 and BSS as control *n* = 3) showed healthy retinal vasculature and a healthy optic disc (B) with intact retinal layers and structure (C). No retinal thinning (D) nor increased IOP (E) was detected. ERG showed intact retinal function during light‐ and dark‐adapted analyses with no clinically relevant differences in amplitudes (F) and latencies (G). (H) showcases exemplary fluorescein‐ and ICG late‐phase images with no signs of leakage or vessel sheathing.

**TABLE 1:** Overview of the in vivo experiments in pigs.

Injected formulation	Number of animals	Injected eye
Liposomes without surface modification	*N* = 3	Injected in the right eye, left eye negative control
0.1% Cyclic‐arginine surface modified	*N* = 3	Injected in the right eye, left eye negative control
0.5% Cyclic‐arginine surface modified	*N* = 3	Injected in the right eye, left eye negative control
1.0% Cyclic‐arginine surface modified	*N* = 3	Injected in the right eye, left eye negative control
Balanced salt solution	*N* = 3	Injected in the right eye, left eye negative control

Post‐mortem histological evaluation showed physiological retinal layering in hematoxylin and eosin (H&E) staining. No structural alterations such as atrophy or change in density were visible (Figure 4A). By immunofluorescent analysis, two markers were analyzed: GFAP to detect Müller‐glia cell activation and CD45 to detect immune cell infiltration. Staining for GFAP showed a physiological distribution of retinal glial cells. In all analyzed eyes, no signs of Müller cell activation could be observed (Figure 4B). Immunofluorescence for CD45 shows tissue resident leucocytes in the choroid of liposome injected, BSS injected and untreated eyes but no sign of additional intraretinal leucocyte infiltration or migration (Figure 4C). Further, as presented in Figure 4D, no increase in aqueous cytokines was apparent between left control eyes and eyes injected with the ARP‐surface modified liposomes.

**FIGURE 4:**
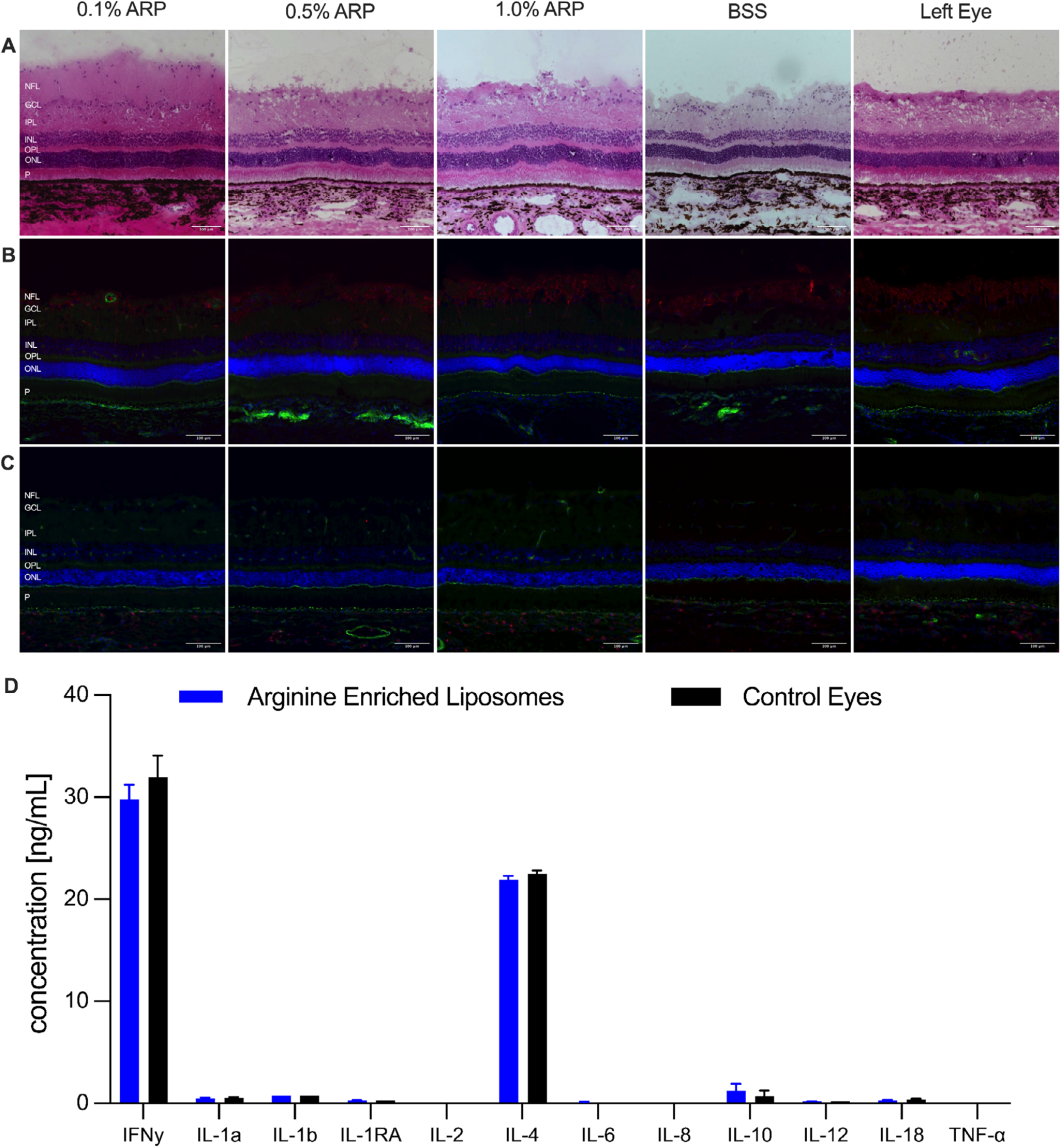
Histological and immunofluorescence staining of the retina of eyes injected with modified liposomes, BSS and an untreated left control eye at 6 weeks after injection. (A) H&E staining shows physiological retinal layering in all eyes. No visible change in retinal structure. (B&C) Immunofluorescent staining of GFAP (red) and CD45 (red), respectively. Additionally, DAPI staining for visualizing nuclei (blue) and Phalloidin staining for f‐actin in cytoskeleton (green) was performed. (B) GFAP staining showed physiological retinal distribution (red) and no visible Müller cell activation in all eyes. (C) Staining for CD45 showed tissue‐resident leucocytes, but no additional intraretinal leucocyte infiltration (red). Magnification x100. (D) No significant differences and specifically no increase in aqueous cytokine levels between treated and control eyes were apparent using a porcine multiplex beads assay (mean and SD presented). Abbreviations: Ch – choroid, ELM – external limiting membrane, GCL – ganglion cell layer, GFAP – glial fibrillary acidic protein, INL – inner nuclear layer, IPL – inner plexiform layer, NFL – nerve fiber layer, ONL – outer nuclear layer, OPL – outer plexiform layer, P – photoreceptor layer.

### 2.4 Visualization of in vivo particle mobility

Particle mobility was analyzed using fundus photographs of the injection site and by OCT images of the preretinal vitreous body at the visual streak. Figure 5A depicts photographs of the injection site for every liposomal formulation. A clear concentration‐dependent relationship between intravitreal particle mobility and surface modification was observed with negligible particle movement being detected at 1% ARP‐surface modification. Figure 5B shows exemplary OCTs at different time points for every injected liposomal formulation. Significant differences in preretinal vitreous haze were apparent and subsequently analyzed.

**FIGURE 5:**
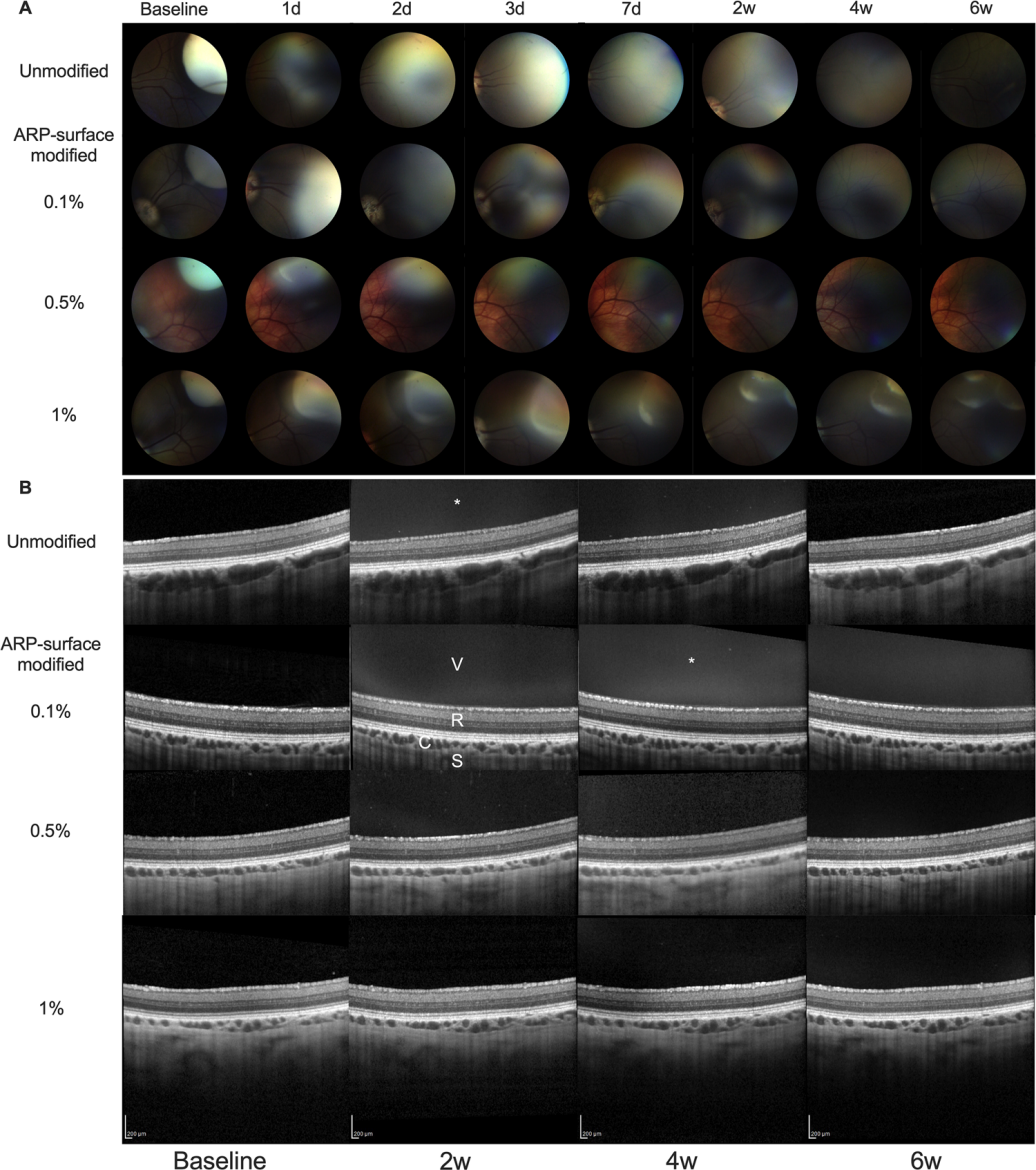
Qualitative visualization of intravitreal particle mobility. Animals underwent fundus photographs immediately after injection as well as 1, 2, 3, and 7 days as well as 2, 4, and 6 weeks after injection. Images were taken from the same region in the temporal superior quadrant in a standardized scheme by the same three examiners. A clear difference in intravitreal mobility was observed. While unmodified liposomes expansively distributed in the vitreous within 2 days, mobility of 0.1% and 0.5% ARP‐modified liposomes was reduced in a concentration‐dependent manner. 1.0% ARP‐modified liposomes showed absence of intravitreal mobility (A). This is further reflected in the appearance of the preretinal vitreous body at the visual streak, the equivalent to the human macular region in pigs (B). These OCT images show a tomography of the sclera (S), the choroid (C), the retina (R) and the preretinal vitreous body (V). As visible on some of the images, vitreous haze is quantifiable through an increase in hyperreflectivity of the preretinal vitreous body (two exemplary images with pronounced hyperreflectivity marked with asterisk). This vitreous haze was subsequently analyzed quantitatively in Figure 6, OCT images are presented here as an overview.

### 2.5 Vitreous Haze Analysis

To allow the quantitative analysis of preretinal vitreous haze, the vitreous body: retinal pigment epithelium ratio (VIT/RPE ratio) was calculated by OCT scans for every individual animal at all time points. Using the follow‐up function of the Spectralis OCT, the exact same scan location was guaranteed at every time point. The vitreous haze analysis uses the retinal pigment epithelium, visualized as a hyperreflective band in the OCT image that is not influenced by the reflectivity of particles or haze in the vitreous cavity, as a stable control. Every OCT image of the visual streak also includes the preretinal vitreous body as visualized in Figure 6A. This preretinal vitreous body was also marked as a region‐of‐interest. Naturally, the vitreous is hyporeflective and optically clear, thus, changes to this parameter are directly linked to the optically active nanoparticular systems in the experiments conducted. The reflectivity of the vitreous was then calculated into a ratio with the stable results of the retinal pigment epithelium. Figure 6A depicts two examples of this process: one showing very low and clinically significant vitreous haze. Based on previous clinical literature, a value of >0.15 was defined as clinically relevant increased vitreous haze expected to cause glare and patient discomfort [44]. Untreated control eyes and eyes injected with BSS showed a stable VIT/RPE‐ratio below <0.15. Regarding liposomal formulations, this was only achieved by the 1%‐ARP modified liposomal formulation. For unmodified liposomal formulation as well as the 0.1% and 0.5%‐ARP modified liposomal formulation a concentration‐dependent increase in vitreous haze was visible. Interestingly, while unmodified liposomes showed the greatest vitreous haze 2 weeks after injection, even low surface modification delayed the peak vitreous haze to 4 weeks (Figure 6B,C). After euthanasia, vitreous bodies were removed from the vitreous cavity and compared. Figure 6D shows exemplary images of the localized liposomal reservoir of 1%‐ARP modified liposomes compared to the diffuse distribution of unmodified liposomes even after 6 weeks.

**FIGURE 6:**
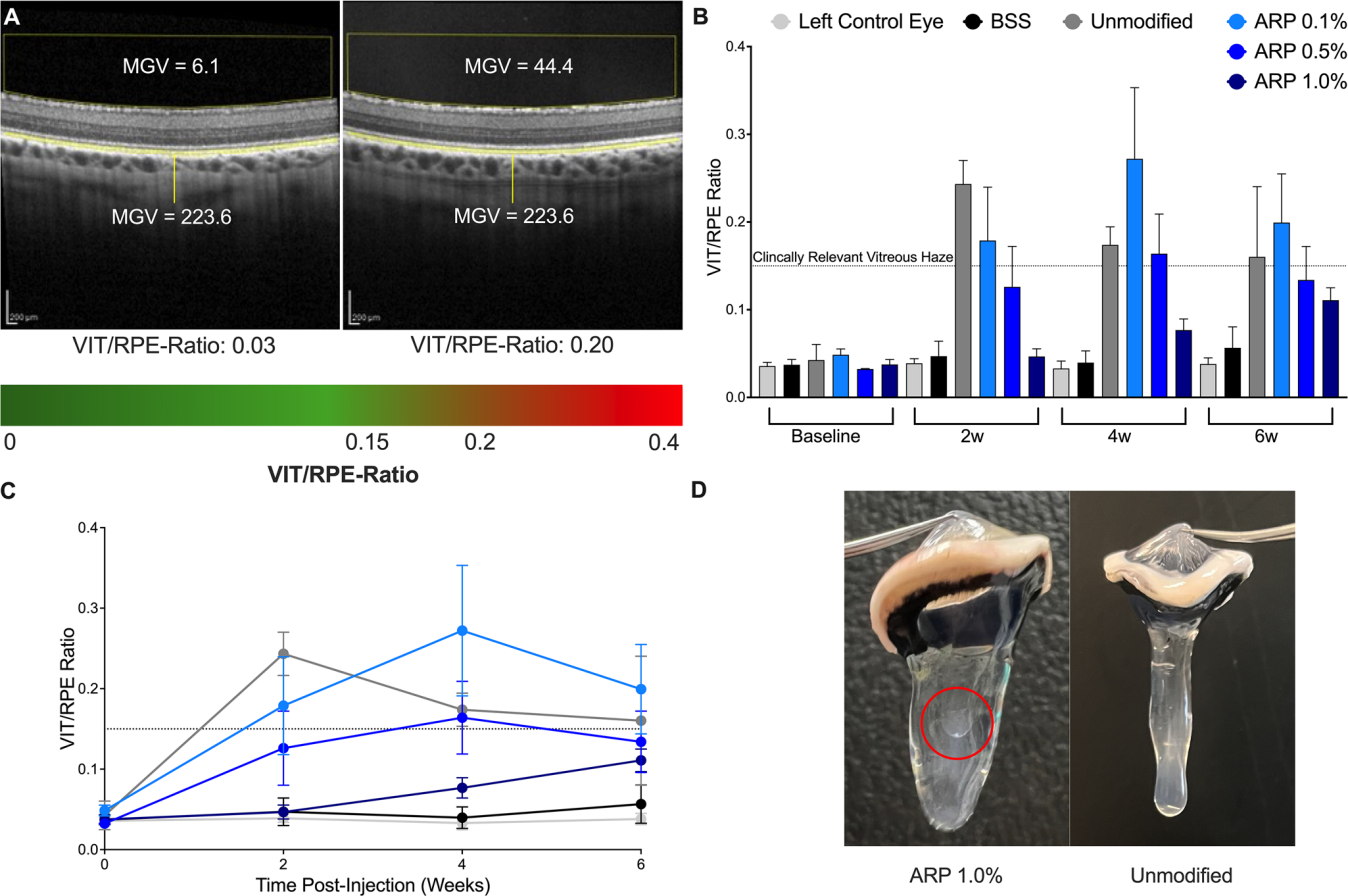
Vitreous haze analysis. Two regions of interest are shown in respective OCT images. The first ROI is placed on the preretinal vitreous body in the visual streak area, the second ROI is placed on the retinal pigment epithelium. Mean gray values are calculated and a ratio is generated as previously demonstrated by Kearse et al. Values of >0.15 generally lead to substantial vitreous haze and were used as the cut‐off for comparison (A). Vitreous Haze (analyzed in a total of 15 animals) remained below clinically relevant values only for the injection of BSS (*n* = 3) and 1.0%‐ARP modified liposomes (*n* = 3) as well as untreated control eyes (*n* = 5, one from each experimental group) (B). The timepoint exhibiting greatest vitreous haze was delayed in all ARP‐modified liposomal groups to 4 weeks (0.1% and 0.5% ARP) or even 6 weeks (1.0% ARP) compared with unmodified liposomes at 2 weeks (C). After euthanasia, the anterior segment and the vitreous were recovered. While the remaining liposomes were concentrated at the injection site in the 1.0% ARP‐group, a diffuse distribution was noted in the unmodified liposome group (D).

## 3 Discussion

In this study, we demonstrate that cyclic‐arginine surface modification systematically modulates the intravitreal mobility of liposomal nanocarriers in vivo. By quantifying intravitreal particle mobility across a controlled series of surface‐modification densities, we show a clear concentration‐dependent decrease in diffusivity, establishing cyclic‐arginine as a tunable determinant of nanoparticle behavior within the vitreous in alive pigs. These findings identify surface engineering, and explicitly not carrier composition, as a tunable parameter to predict mobility in the vitreous body in a large animal in vivo model over 6 weeks, all while proving excellent biocompatibility.

The mechanistic interpretation points toward electrostatic and matrix‐interaction effects. The vitreous is dominated by a negatively charged macromolecular network [45, 46]. An increasing density of cyclic‐arginine‐modified phospholipids on the liposome's surface progressively shifts surface interactions from weak adhesion toward lower net repulsion and increased steric hindrance, thus decreasing nanoparticular movement in a concentration‐dependent fashion. This hypothesis is further supported by the demonstrated concentration‐dependent decrease in zeta potential in the presence of HA, indicating its absorption on the liposomal surface. This concentration‐response relationship suggests a threshold at which cyclic‐arginine sufficiently inhibits intravitreal mobility, followed by a plateau where further surface modifications yield diminishing returns. This aligns with concepts known from cell‐penetrating peptides utilized in the setting of easing intracellular uptake where concentrations higher than 2 mol% lead to diminished returns and compromised particle characteristics, most likely due to interparticle interactions [47, 48]. While not examined in‐depth, the diminished returns at greater concentrations are not toxicity‐related but rather through the compromised particle characteristics. Previous studies have investigated single particle movement in vitreous or vitreous‐like media showing that cationic surface charge can lead to immobilization [36, 49]. However, for the first time, a quantitative evaluation of this surface modification not to allow intracellular uptake but to gradually inhibit mobility in the eye in vivo in a translational large animal model was established.

Very few studies have characterized intravitreal diffusion of nanocarriers and did so largely in ex vivo gels or simplified in vitro vitreous analogs [36, 50]. These systems capture only parts of the physiological ultrastructure and often overestimate mobility, as the vitreous body quickly deteriorates and liquifies ex vivo [45, 46]. In contrast, this study and the in vivo model applied captures the real viscoelastic and biochemical environment, including temperature, ionic composition, proteoglycan density, and micro‐heterogeneity. To our knowledge, no previous study has provided a direct in vivo concentration–effect curve for surface‐modified liposomes, making our dataset uniquely suited to inform translational drug‐delivery design. Previously, the only in vivo study of surface‐modified nanocarriers was conducted by Nieto et al. [51] However, the particles examined were porous silicone microparticles of a size of around 100 µm, thus, with the average pore size of the vitreous body expected to be 1 µm or below, results are not translatable to nanoparticles with a size in the nanometer range [51]. Further, the authors did not study vitreous haze or intravitreal mobility using repetitive fundus photography as it was not their primary interest. As such surface modifications were extensively tested in vitro, our study shows the great benefit in delivering a strong multimodal biocompatibility examination with excellent results in line with previous data [14].

These findings are crucial for designing ocular therapeutics. Modulating mobility may enable spatially controlled drug distribution within the vitreous and may help to optimize targeting of posterior structures, such as specific parts of the retina prone to develop proliferative vitreoretinopathy or delivering neuroprotective compounds directly to optic nerve head. For sustained‐release formulations, appropriately tuned mobility is critical for balancing local retention with effective tissue exposure while minimizing visual disturbance. Cyclic‐arginine therefore represents a modular strategy applicable to a wide range of nanoparticle systems, including drug‐loaded liposomes and various polymeric carriers, such as poly‐lactic acid nanoparticles [52]. Its tunability may support the development of more predictable and patient‐specific delivery systems. Additionally, the retina is shielded from the vitreous cavity through the internal limiting membrane (ILM). The ILM acts as a molecular sieve with a cutoff of around 30 nm [53] and thus should prevent direct diffusion of the liposomal formulations directly into retinal tissue. Thus, while retinal cellular internalization was not directly evaluated, any uptake is rather unlikely. Further, the cationic surfaces in the vitreous cavity did not show any negative effects for the duration of 6 weeks and are undergoing degradation and clearance [36, 49].

Our study shows several strengths, especially in vivo tracking, a well‐defined concentration gradient, and the use of a single carrier platform to isolate and solely study the effects of this distinct surface modification. Control groups with unmodified liposomes and sham‐injections with BSS only, as well as control eyes not undergoing any intervention are in place and strongly support the results obtained. One important advantage of this platform technology is its scalability. As proven by several approved liposomal applications, specifically through lyophilization, stability and scalability can be achieved at a large scale. However, some limitations should be acknowledged. Species‐specific differences in vitreous composition may limit direct translation to humans, however, of all animal models, pigs are continuously gaining interest and are now often referred to as the closest model apart from non‐human primates in which many trials are not feasible nor ethically supported. A porcine model was chosen over rodents and rabbits because of its anatomical and physiological similarities to the human eye. With a cone‐enriched visual streak, preserved intraretinal vasculature and a similar ERG response, the porcine model gained great popularity over the last years as it is more accessible than non‐human primates and more accurate than more traditional models. The observation window, while sufficient for mobility quantification, does not capture long‐term redistribution or clearance and longer‐term studies in pig breeds with a more acceptable weight growth curve should be conducted in the future. However, with landrace pigs and their exponential weight growth curve, a longer study duration is not physically feasible. Besides, biocompatibility was not evaluated in this study after repetitive injection. However, as remnants of the first injection are still detectable after 6 weeks, continuous exposure was still given. Further, future studies should focus on the impact of nanocarrier diffusion on intravitreal pharmacokinetics and pharmacodynamics. Based on previous pharmacokinetics data in large animal models for small molecule drugs, this is a specifically promising future approach. Potential target compounds should therefore include methotrexate and tyrosine kinase‐inhibitors. Specifically for methotrexate, relevant release from liposomal formulations has been proven for over 6 weeks [14] and thus a meaningful duration that could be modulated with the here presented surface modification. Finally, further studies should illuminate the impact of ARP‐surface modifications on other nanocarrier formulations [54] and other ways of administration.

## 4 Conclusion

Our findings establish cyclic‐arginine surface engineering as a highly biocompatible, controllable, and concentration‐dependent regulator of intravitreal nanoparticle mobility in vivo in a translational large animal model. This concept paths the way for designing next‐generation ocular drug‐delivery systems with predictable spatial behavior and improved translational potential.

## 5 Experimental Section

### 5.1 Preparation of Cyclic‐Arginine Modified Liposomes

Liposomes were prepared by thin film method and dual centrifugation using a Zentrimix 380 R (Hettich GmbH & Co. KG, Tuttlingen, Germany) as described previously [34]. Soy lecithin (Lipoid GmbH, Ludwigshafen am Rhein, Germany), cholesterol (Sigma Aldrich, Taufkirchen, Germany), and ARP‐phospholipid conjugate (synthesized as described previously [48]) were used for liposome formulation. First, the lipids and the ARP‐conjugate were dissolved in a mixture of chloroform and methanol (Sigma Aldrich, Taufkirchen, Germany) (9:1 v/v, soy lecithin and cholesterol = 100 mM; ARP‐conjugate = 5 mM), and mixed according to the respective liposomal compositions, as shown in Table S1. Organic solvents were evaporated under a nitrogen stream. Lipid films were dried in a vacuum chamber for 1 h. Prior to dual centrifugation, 152 mg of Yttria‐stabilized zirconia ceramic beads (Sigmund Lindner GmbH, Warmensteinach, Germany) were added, and dual centrifugation was performed in two runs by addition of the required volumes of aqueous media. Subsequently, 133 µL aqueous media was added and the liposomes were vortexed shortly.

### 5.2 Characterization of Cyclic Arginine‐Modified Liposomes

#### 5.2.1 Dynamic Light Scattering

The average particle size and PDI were measured in triplicates by dynamic light scattering at room temperature as well as zetapotential using the automatic mode of a Zetasizer Ultra from Malvern^TM^. For size (z‐average) and PDI determination, liposomes were diluted with PBS to a final lipid concentration of 0.01% (v/v). Zeta potential measurements were performed after diluting the liposomal formulations with 10% PBS to a concentration of 1.9 mg/mL of the respective liposomal formulation. The default settings of the automatic mode of the Zetasizer Ultra are presented in the Supporting Information (Table S2). The alteration of the zeta potential was evaluated by incubating the respective liposomal formulation in 2% HA (11 kDa) dissolved in 10% PBS.

#### 5.2.2 Cryo‐TEM Analysis of Liposomal Morphology

Liposomes were applied to holey carbon‐coated grids (Lacey, Tedpella, USA) initially glow‐discharged during 45 s at 20 mA in GloCube Plus (Quorum, UK). After 10 s of incubation at 10°C and 90% humidity, grids were blotted from the back side using grade 1 filter paper and then vitrified in liquid ethane at −180°C with a Leica GP2 plunger (Leica Microsystems, Vienna, Austria). These vitrified grids were transferred to a Talos 200 electron microscope (FEI, Hillsboro, OR, USA) with a Gatan 626 cryo‐holder (Gatan, Pleasanton, USA). Electron micrographs were acquired at an accelerating voltage of 200 kV using a low‐dose system (40 e‐ Å‐2), while maintaining the sample at −175°C. Images were captured on a 4 K × 4 K Ceta CMOS camera.

#### 5.2.3 In Vitro Cytotoxicity of Liposomal Formulations in ARPE‐19 Cell Culture

ARPE‐19 cells (LGC Standards GmbH, Wesel, Germany), a human retinal pigment epithelial cell line, were cultivated in Dulbecco's modified Eagle's medium containing nutrient mixture F12 (1:1) (DMEM‐F12) (Gibco/BRL Life Technologies, Grand Island, NY) supplemented with 10% fetal bovine serum (Bio and Sell GmbH, Feucht, Germany), 15 mM HEPES and a combination of penicillin and streptomycin (100 U mL‐1, 100 µg mL‐1) and incubated at 37°C and 5% CO_2_. Supplements were purchased from Fisher Scientific, Schwerte, Germany. Cells were split upon reaching ≈90% confluence. For the viability assay, cells at a density of 1 × 10^4^ cells per well were seeded into a 96‐well microplate (Costar Assay Plate, Corning Inc., NY, United States) and cultured for 24 h as described above. After incubation, liposomes with 0.1% ARP, 0.5% ARP, or 1% ARP were serially diluted in cell medium and added to the cells to reach final concentrations ranging from 0.005 to 2.5 mg mL‐1 lipid. Cells were incubated with the liposomes as described above for another 24 h. PrestoBlue HS Cell Viability Reagent (Invitrogen, Life Technologies, Darmstadt, Germany) was added to the wells to obtain a final concentration of 10%, and the plates were incubated at 37°C and 5% CO_2_ for 2 h. Afterwards, fluorescence was measured at 590 nm with excitation at 540 nm using an Infinite F200 PRO microplate reader (Tecan, Männedorf, Switzerland). Cell viability was calculated using the untreated control cells as a reference for 100% viable cells.

### 5.3 Animal Studies in the Porcine Model

Fifteen male German Landrace pigs were examined and housed at the Interfaculty Institute for Biomedical Research at Heidelberg University. The animals were housed at 20 ± 2°C with a 12/12 h light/dark cycle with restricted access to food and free access to water. Environmental enrichment included chains, biting sticks, and straw bedding. All experiments adhered to regional, national, and European laws and standards for experimental animal welfare. Table 1 showcases the exact experiments individual animals underwent.

All treatments and examinations took place under general anesthesia after previous sedation. Initially, 220 mg of azaperone (Sedanol 40 mg/mL, WDT, Germany) were injected intramuscularly, followed by another intramuscular injection of 400 mg of ketamine (Ketamin 100 mg/mL, CP‐Pharma, Germany) and 30 mg of midazolam (Midazolam 5 mg/mL, Hameln Pharma, Germany) after 20 min. Once fully sedated, animals were intubated and anesthesia sustained with 2 vol.% inhalation of isoflurane (Isofluran CP 1 ml/ml, CP‐Pharma, Germany).

#### 5.3.1 Intravitreal Injections

Following baseline pressure measurements and biocompatibility assessments, intravitreal injection was performed in the right eye through the pars plana of the ciliary body, using a 30G needle (BD Microlance, Becton Dickinson NJ, United States) and a 0.2 ml syringe (SJJ Solutions, Netherlands). Prior to injection, eyes were disinfected with povidone‐iodine (Braunol, B. Braun SE, Germany). Injections were performed by two trained ophthalmologists (MH and NJ), ensuring adherence to best clinical practice. All injections were placed in the temporal superior quadrant of the eye to allow interindividual comparison. Afterwards, the eye was disinfected again using povidone‐iodine. Moxifloxacin eye drops (VIGAMOX, Novartis Pharma, Switzerland) and ofloxacin ointment (Floxal 3mg/g, Bausch & Lomb, Canada) were applied and the eye was covered until the animal regained full consciousness.

#### 5.3.2 In Vivo Biocompatibility Examination

Both, left and right eyes, were examined at baseline before injection and at 2, 4, and 6 weeks post‐injection. Additionally, fundus photography was performed at 24 h, 48 h, 72 h, and 1 week post‐injection, to closely monitor intravitreal distribution of the liposomes shortly after treatment. To assess biocompatibility, a previously established protocol by Hammer et al. [14], including IOP, OCT, fundus photography, and ERG (Figure 3A) was applied. While right eyes served as the experimental group, left control eyes were used as intraindividual controls not undergoing IVIs. Prior to the examination, eyes were locally anesthetized using Oxybuprocaine (Conjucain EDO, Bausch & Lomb, Canada) and the pupils dilated with Cyclopentolate (Zyklolat EDO, Bausch & Lomb, Canada), Tropicamide (Mydriaticum Stulln DU, Pharma Stulln, Germany), and Phenylephrine (Neosynephrin POS 5%, Ursapharm Arzneimittel, Germany) until the pupil showed maximal dilation. Retinal integrity and structure were evaluated using OCT (SPECTRALIS Flex OCT, Heidelberg Engineering, Germany), applying a 20° dense scan with a 30° infrared reflectance image. To ensure comparable follow‐up, the follow‐up function was employed to record the exact location in the visual streak. IOP was evaluated using the iCare TonoVet Plus (Icare Finland Oy, Finland). Additionally, the posterior pole of the eye, including the optic nerve head, was photographed with a fundus camera (ClearView2, Optibrand, CO, United States). ERG recordings were performed on both eyes of the pigs at all time points. To ensure protection of the cornea and stable electrical contact, the corneal contact lens electrode (ERG‐Jet, Fabrinal SA, Switzerland) was placed on the eye with a Carbomer 980 Gel (Vidisic, Bausch & Lomb, Canada). Intradermal electrodes (Natus Ultra Subdermal Needle Electrode, Natus Neurology Inc., WI, United States) were used, with the reference electrode placed 3 cm temporal of the lateral canthus of the examined eye and the ground electrode centered on the forehead between both eyes. A handheld ERG‐device (RETevet, LKC Technologies, MD, United States) produced full‐field (Ganzfeld) light stimuli and recorded the subsequently evoked potentials. The device was previously successfully used to characterize ERG responses in landrace pigs [55, 56]. Testing followed a pre‐installed protocol, based on the ISCEV Standard for full‐field clinical ERG [57]. Before dark adapted tests, eyes were dark‐adapted for 15 min. At the final biocompatibility examination, fluorescein and ICG was performed using the Spectralis Flex Platform.

#### 5.3.3 Cytokine Analysis

To quantify inflammation, levels of GM‐CSF, IFNγ, IL‐1α, IL‐1β, IL‐1ra, IL‐2, IL‐4, IL‐6, IL‐8, IL‐10, IL‐12, IL‐18, and TNFα were measured in aqueous taps using the MILLIPLEX Porcine Cytokine/Chemokine Magnetic Bead Panel (Sigma Aldrich, Merck, Germany) kit. Aqueous taps were taken after euthanasia. The kit was used following manufacturer's instructions.

#### 5.3.4 Vitreous haze analysis

Vitreous haze has been quantified by its signal intensity in relation to that of the retinal pigment epithelium (RPE), as it has been previously been described by Keane and colleagues [44, 58]. For each individual, five B‐scans of the visual streak were selected from baseline imaging. Utilizing the follow‐up function of the Spectralis OCT device, the exact location and its corresponding scans were analyzed at 2, 4, and 6 weeks.

To compare the vitreous to the RPE, two regions of interest (ROIs) were defined for each B‐scan (Figure 6A). First, the pictured vitreous, ranging from the ILM to the anterior limit, second, the RPE, which was manually marked for each scan.

Since not every scan displayed the same area of vitreous, the vitreous ROI was limited by the scan showing the least amount of vitreous, which defined the anterior limit of the vitreous ROI.

B‐scans were directly exported from the HEYEX software (Heidelberg Engineering, Heidelberg, and Germany) and imported into ImageJ [59]. Scans were processed as 16‐bit images and the mean gray value (MGV) of ROIs was measured. Ultimately, the MGV of the vitreous ROI was divided by the MGV of the RPE ROI, resulting in a “haze‐ratio”, representative of the vitreous’ signal intensity. The haze‐ratio of all individuals of one group was averaged for each time point and subsequently statistically compared.

#### 5.3.5 Histopathological Evaluation of the Retina

Immunostainings and H&E staining of all right, experimental eyes and one left eye from each test group were performed. Specifically, a staining for GFAP to detect glial cell proliferation and CD45 to detect immune cell infiltration, both signs of possible subclinical inflammation, was performed. A detailed description of the methodology used is presented in the Supporting Information.

### 5.4 Statistical Analysis

Retinal thickness was compared using a restricted maximum likelihood (REML) mixed‐effects model with time as a within‐subject factor and treatment as between‐subject factor, and subject included as a random effect. Sphericity was not assumed, and the Geisser‐Greenhouse correction applied where appropriate. For comparing IOP, ERG amplitudes and latencies of treated and control eyes, paired t‐tests were used and the Holm‐Šídák correction utilized where applicable to account for multiple testing. P‐values <0.05 were considered statistically significant. Data is presented as mean ± SEM unless otherwise specified. Statistical analyses were performed using Prism 10.4.1 (GraphPad Prism, San Diego, CA, United States).

## Author Contributions

**Maximilian Hammer**: conceptualization, investigation, funding acquisition, writing – original draft, writing – review and editing, visualization, validation, methodology, software, formal analysis, data curation, supervision. **Bryan Calder Ackermann**: investigation, writing – review and editing, data curation, visualization, writing – original draft, formal analysis, validation. **Anna Stoß**: investigation, writing – review and editing, formal analysis, data curation. **Niklas Junker**: investigation, writing – review and editing, formal analysis, data curation, validation. **Ludwig Geisweid**: investigation, writing – review and editing, formal analysis, data curation. **Sabrina Wohlfart**: investigation, visualization, writing – review and editing, methodology, data curation, formal analysis, project administration. **Jonathan Herth**: investigation, writing – review and editing, formal analysis, data curation. **Simon William Pohl**: investigation, writing – review and editing, formal analysis, data curation. **Manuel Ben Böhmann**: investigation, writing – review and editing, formal analysis, data curation. **André Schulz**: writing – review and editing, methodology, visualization, formal analysis, supervision. **Victor Aristide Augustin**: conceptualization, investigation, writing – review and editing, methodology, project administration, supervision. **Gerd Uwe Auffarth**: conceptualization, writing – review and editing, methodology, project administration, supervision, resources. **Philipp Uhl**: conceptualization, funding acquisition, writing – review and editing, writing – original draft, methodology, project administration, supervision, resources.

## Funding

MH is supported by the Dr. Werner Jackstädt Award to the Retinologische Gesellschaft. This study was supported by the innovate! Academy of the Joachim Herz Foundation directed to MH. MH is supported by the Add‐On Fellowship of the Joachim Herz Foundation. This study was supported by the Ernst und Berta Grimmke Foundation (MH & PU).

## Conflicts of Interest

Philipp Uhl is co‐inventor on patents describing various in vivo applications of arginine‐modified liposomes.

## Supporting information




**Supporting File**: adhm71531‐sup‐0001‐SuppMat.docx.

## Data Availability

The data that support the findings of this study are available from the corresponding author upon reasonable request.
