# Supplementary material for: Engineering Concentration‐Dependent Intravitreal Mobility via Cyclic Arginine‐Enriched Nanocarrier Surface Modification: In Vivo Proof‐of‐Concept in a Porcine Large Animal Model
Source: Adv Healthc Mater. 2026 Aug 5;15(39):e71531. doi: 10.1002/adhm.71531 (PMC13691387; doi:10.1002/adhm.71531)
Supplement: Supplementary file 1 [file ADHM-15-0-s001.docx]

Supporting Information

Engineering concentration-dependent intravitreal mobility *via* cyclic arginine-enriched nanocarrier surface modification: *in-vivo* proof-of-concept in a porcine large animal model

Maximilian Hammer, Bryan Calder Ackermann, Anna Stoß, Niklas Junker, Ludwig Geisweid, Sabrina Wohlfart, Jonathan Herth, Simon William Pohl, Manuel Ben Böhmann, Andre Schulz, Victor Aristide Augustin, Gerd Uwe Auffarth, Philipp Uhl*

Results:

Nanocarrier composition and particle characteristics (belongs to: Liposome Characterization)

|  | **Ratio [mol-%]** | **Size [nm]** | **PDI** | **Zeta Potential [mV]** | **Zeta Potential after incubation with HA [mV]** |
| --- | --- | --- | --- | --- | --- |
| **Non-modified liposomal nanocarriers** | | | | |  |
| **Lecithine** | 90 | 106.90 ± 10.0 | 0.23 ± 0.07 | -3.84 ± 0.35 | 0.18 ± 0.74 |
| **Cholesterol** | 10 |  |  |  |  |
| **0.1% ARP-liposomal nanocarriers** | | | | |  |
| **Lecithine** | 89.9 | 127.10 ± 4.90 | 0.22 ± 0.03 | -1.39 ± 0.46 | -2.72 ± 0.96 |
| **Cholesterol** | 10 |  |  |  |  |
| **ARP** | 0.1 |  |  |  |  |
| **0.5% ARP-liposomal nanocarriers** | | | | |  |
| **Lecithine** | 89.5 | 116.20 ± 1.90 | 0.24 ± 0.01 | 4.63 ± 0.85 | -8.93 ± 2.94 |
| **Cholesterol** | 10 |  |  |  |  |
| **ARP** | 0.5 |  |  |  |  |
| **1.0% ARP-liposomal nanocarriers** | | | | |  |
| **Lecithine** | 89 | 133.60 ± 10.70 | 0.28 ± 0.06 | 14.17± 2.78 | -12.39 ± 2.80 |
| **Cholesterol** | 10 |  |  |  |  |
| **ARP** | 1 |  |  |  |  |

**Table S1: Liposome characteristics without and with 0.1%, 0.5% and 1%-ARP surface modification and after incubation with hyaluronic acid (HA; 2%, 11 kDa).** While size of the liposomes is not strongly influenced, the effect of the ARP conjugation is clearly visible in the increasingly positive surface charge as evidenced by increased zeta potential. After incubation with HA the zeta potential reversed towards negative values, indicating adsorption of HA onto the liposomal surface. Mean ± SD is shown.

**Methods:**

**Details for the histopathological evaluation of the retina (belongs to: Histopathological evaluation of the retina)**

After euthanization of animals, the right eyes and one left eye from each test group were enucleated and immediately processed. For histological preparation, the anterior chamber section was removed by making an incision along the pars plana with a scalpel (B. Braun SE, Melsungen, Germany). After removing the anterior chamber section, the vitreous body was removed using scissors and forceps. The posterior eyecup was then cooled in isopentane (>99%, Carl-Roth GmbH + Co. KG, Karlsruhe, Germany) pre-cooled with liquid nitrogen and subsequently frozen in liquid nitrogen. Additionally, the posterior eyecup was filled with freezing medium (Tissue freezing medium, LEICA Biosystems, Nußloch, Germany), frozen in liquid nitrogen, and stored at -80 °C. For histological sectioning, 5-7 µm thick slices were cut using a cryotome (Leica CM1850, LEICA Biosystems, Nußloch, Germany), cooled to -16 to

-18 °C and mounted on microscope slides (SuperFrost PlusTM, Epredia, Portsmouth, NH, USA) which were then stored at -80 °C.

For Immunofluorescence, the slides were defrosted and subsequently fixed in pre-cooled acetone (>99,5%, Carl-Roth GmbH + Co. KG, Karlsruhe Germany) at -20 °C. After drying, each slice was circled with a hydrophobic barrier pen (Dako® PAP Pen, Agilent Technologies, Santa Clara, USA) and 10% horse serum (S-2000, Vector Laboratories, Burlingame, USA) diluted in 1% PBS was applied for 30 minutes at room temperature in a dark humid chamber to block unspecific protein binding. After removing the horse serum, anti-CD45 (clone K252.1E4, Bio-Rad Laboratories, Hercules, CA, USA) and anti-GFAP (clone GF 12.24, PROGEN, Heidelberg, Germany) antibodies were applied at a 1:100 dilution in PBS. For negative controls, PBS was used instead of the primary antibodies. The slides were then incubated overnight at -4 °C in a dark humid chamber. After incubation, the slides were washed twice for 5 minutes each in PBS. Next, the secondary antibodies and fluorochromes were added. DAPI (D9542, Sigma-Aldrich, Merck, Germany), Cy3 conjugated donkey anti-mouse secondary antibody (Jackson ImmunoResearch Laboratories, West Grove, USA) and Phalloidin (CytoPainter, ab176753, Abcam, Cambridge, UK) were applied together at dilutions of 1:1000, 1:200, and 1:100 in PBS, respectively. After incubation for 30 minutes at room temperature in a dark humid chamber, the slides were washed again twice for 5 minutes each, covered from light to prevent fluorochrome fading. After washing the slides were mounted with coverslips (24 x 50 mm, borosilicate glass, VWR international, Radnor, USA) using vecta-mount (Vector Laboratories, Burlingame, USA).

For H&E staining, the slides were defrosted, dried, and then rinsed with distilled water. They were subsequently immersed in hematoxylin solution (acidic Mayer´s hematoxylin solution, Carl-Roth GmbH + Co. KG, Karlsruhe Germany) for 6 minutes and then blued under running tap water for 15 minutes. After rinsing the slides again in distilled water, they were counterstained using eosin G solution (0.5%, Carl-Roth GmbH + Co. KG, Karlsruhe Germany), supplemented with glacial acetic acid (99.8 – 100%, Bernd Kraft GmbH, Den Haag, Netherlands), for 3 minutes. Following the eosin G solution, the slides were rinsed with tap water and then passed through ascending concentrations of ethanol. They were immersed in 70%, 96%, and 100% ethanol (all Zentralbereich Neuenheimer Feld, Heidelberg, Germany) for 5 minutes each. Afterwards the slides were placed in ROTHIHistol (Carl-Roth GmbH + Co. KG, Karlsruhe Germany) for five minutes and then covered with coverslips (24 x 50 mm, borosilicate glass, VWR international, Radnor, USA) using ROTHIHistokitt (Carl-Roth GmbH + Co. KG, Karlsruhe Germany).

The Nikon Eclipse Ni-E microscope from the Nikon Imaging Center in Heidelberg was used for imaging, with a magnification of x100.

**Default settings of the automatic mode of the Zetasizer Ultra (belongs to: Characterization of cyclic -arginine- modified liposomes):**

For all liposomal investigations, the automatic mode of the Zetasizer Ultra from Malvern™ (Malvern Instruments Ltd., Worcestershire, United Kingdom) was used: number of measurements = 3; number of runs = 5 (size, PDI) or 50 (zeta potential); equilibration time = 60 s; refractive index solvent 1.330; refractive index polystyrene cuvette 1.590; viscosity = 0.8872 mPas; temperature = 25 °C; dielectric constant = 78.5 Fm-1; Smoluchowski equation.

| Run | Time [min] | Aqueous volume [µL] |
| --- | --- | --- |
| 1 | 15 | 27 |
| 2 | 5 | 90 |
| vortex |  | 133 |

**Table S2. Settings of the dual centrifugation for liposomal formulations.** Run 1 and 2 were performed by dual centrifugation at room temperature (20 °C) at 2.500 rpm, 5/9.

**Figure S1. Respective Cryo-TEM images of the remaining liposomal formulations evaluated.**

All formulations showed a predominantly unilamellar and univesicular morphology in cryo-TEM imaging. Cryo-TEM of the 1% ARP-modified liposomal formulation is presented in the main manuscript, Figure 2D.

**Figure S2. ERG measurements of animals receiving unmodified liposomes and modified liposomes with different ARP-concentrations**. Shown are amplitudes and latencies at 6 weeks of the treated eye vs. the left control eye. In line with the summarized data in Figure 3F, no clinically significant differences between the treated and control eye could be observed. For each treatment group, multiple paired t-tests were performed and adjusted p-values after Holm-Šídák correction are displayed in the respective plots. N=12 (n=3 for each group), mean and standard deviation are shown.
